# Age-dependent changes in TDP-43 levels in a mouse model of Alzheimer disease are linked to Aβ oligomers accumulation

**DOI:** 10.1186/1750-1326-5-51

**Published:** 2010-11-11

**Authors:** Antonella Caccamo, Andrea Magrí, Salvatore Oddo

**Affiliations:** 1 Department of Physiology and The Barshop Institute for Longevity and Aging Studies, University of Texas Health Science Center at San Antonio, 7703 Floyd Curl Drive, San Antonio, TX 78229-3900, USA

## Abstract

**Background:**

Transactive response DNA-binding protein 43 (TDP-43) is the pathological protein found in frontotemporal lobar degeneration with ubiquitin positive inclusions and in amyotrophic lateral sclerosis. In diseased tissue, TDP-43 translocates from its physiological nuclear location into the cytoplasm, where it accumulates. Additionally, C-terminal fragments of TDP-43 accumulate in affected brain regions and are sufficient to cause TDP-43 mislocalization and cytoplasmic accumulation *in vitro*. TDP-43 also accumulates in 30% of Alzheimer disease (AD) cases, a finding that has been highly reproducible. The role of TDP-43 in AD and its relation with Aβ and tau pathology, the two neuropathological hallmarks of AD, remains to be elucidated.

**Results:**

Here we show that levels of TDP-43 and its ~35 kDa C-terminal fragment are significantly increased in the 3×Tg-AD mice, an animal model of AD that develops an age-dependent cognitive decline linked to the accumulation of Aβ and tau. We also report that the levels of TDP-43 and its C-terminal fragment correlate with the levels of soluble Aβ oligomers, which play a key role in AD pathogenesis. Notably, genetically reducing Aβ_42 _production restores the levels of TDP-43 and its ~35 kDa C-terminal fragment to control levels.

**Conclusions:**

These data suggest a possible relation between Aβ oligomers and TDP-43.

## Background

Alzheimer disease (AD) is the most common cause of dementia among the elderly [1]. Clinical symptoms include memory loss and impairments in other domains that interfere with mood, reason, judgment, and language [2-4]. Two hallmark neuropathological lesions of AD include the aberrant accumulation of the amyloid-β peptide (Aβ) and neurofibrillary tangles (NFTs) [5]. Other prominent changes include intraneuronal Aβ accumulation, mitochondrial dysfunction, oxidative damage, and changes in the protein quality system [6-9]. Aβ is the primary protein component of amyloid plaques and originates via proteolysis from the amyloid precursor protein [10,11]. Aβ has been the central focal point of AD research for more than a decade and is generally considered the upstream causative factor for AD [12]. The strongest evidence for this position is derived from molecular genetic studies of the three genes (amyloid precursor protein, presenilin 1, and presenilin 2) that underlie familial AD cases, as they all modulate some aspect of Aβ metabolism, increasing the propensity of Aβ to aggregate [13-16]. Indeed, Aβ is an aggregation-prone peptide, and it exists in different forms such as monomers, oligomers, and fibrils [17,18]. In the past few years, *in vitro *and *in vivo *studies have shown soluble Aβ oligomers to be the major neurotoxic species for neurons [19].

The major component of NFTs is the microtubule-associated protein, tau [20-23]. In its normal state, tau is a soluble protein whose function is to promote microtubule assembly and stabilization. Pathological tau protein, by contrast, exhibits altered solubility properties, forms filamentous structures, and is abnormally phosphorylated at specific residues [20-23]. Recent evidence indicates that the accumulation of soluble, phosphorylated tau may be more toxic than NFTs [24-27].

Transactive response DNA-binding protein 43 (TDP-43) is a nuclear protein involved in exon skipping and alternative splicing [28]. The full length fragment has an approximate molecular weight of ~44 kDa. Recently, TDP-43 has been found to be the main protein that accumulates in frontotemporal lobar degeneration with ubiquitin positive inclusions (FTLD-U) and in amyotrophic lateral sclerosis (ALS) [29]. Pathological TDP-43 is mislocalized from the nucleus to the cytoplasm where it accumulates [29]. Additionally, TDP-43 C-terminal fragments have been isolated from affected brain regions [29,30], and their expression *in vitro *is sufficient to cause TDP-43 mislocalization [31-33], suggesting that these fragments may play a role in the disease pathogenesis.

In addition to ALS and FTLD-U, TDP-43 positive inclusions are present in Parkinson disease, dementia with Lewy bodies, and in 30% of AD cases [34-37]. The specific role of TDP-43 in AD has not been identified yet; specifically, it is not clear whether there is a link between TDP-43, Aβ and tau pathology. In this study, we address the relation between Aβ, tau and TDP-43 in the 3×Tg-AD mice, an animal model of AD that develops Aβ and tau pathology, with a temporal- and regional-specific profile that closely mimics their development in the human AD brain [38].

## Results

**Figure 1:**
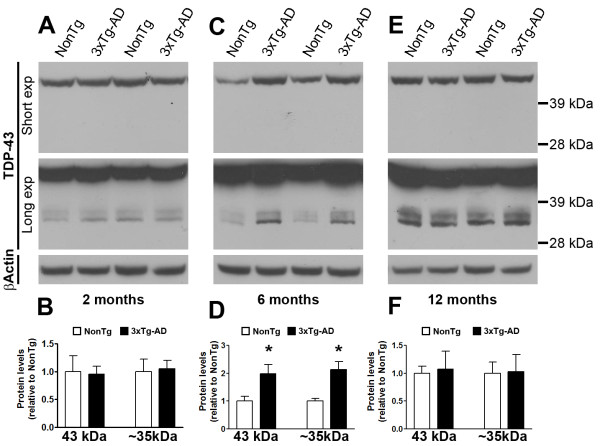
**3×Tg-AD mice have higher levels of TDP-43 and TDP-35**. **(A) **Representative Western blots of proteins from the low salt fraction extracted from 2-month-old 3×Tg-AD and NonTg mice and probed with the indicated antibodies. A longer exposure time was necessary to see the less abundant low molecular weight fragments. **(B) **Quantitative analysis of the blots shows that at 2 months of age the levels of TDP-43 and TDP-35 were similar between 3×Tg-AD and NonTg mice (n = 6/genotype). **(C) **Representative Western blots of proteins from the low salt fraction extracted from 6-month-old 3×Tg-AD and NonTg mice. **(D) **Quantitative analysis of the blots shows that at 6 months of age the levels of TDP-43 and TDP-35 were significantly increased (p = 0.04 and p = 0.01, respectively) in the brains of the 3×Tg-AD mice compared to age- and gender-matched NonTg mice (n = 6/genotype). **(E) **Representative Western blots of proteins from the low salt fraction extracted from 12-month-old 3×Tg-AD and NonTg mice (n = 6/genotype). **(F) **Quantitative analysis of the blots shows that at 12 months of age the levels of TDP-43 and TDP-35 were similar between 3×Tg-AD and NonTg mice (n = 6/genotype). Abbreviation: exp = exposure. Data are presented as ± SEM and analyzed by t-test analysis.

**Figure 2:**
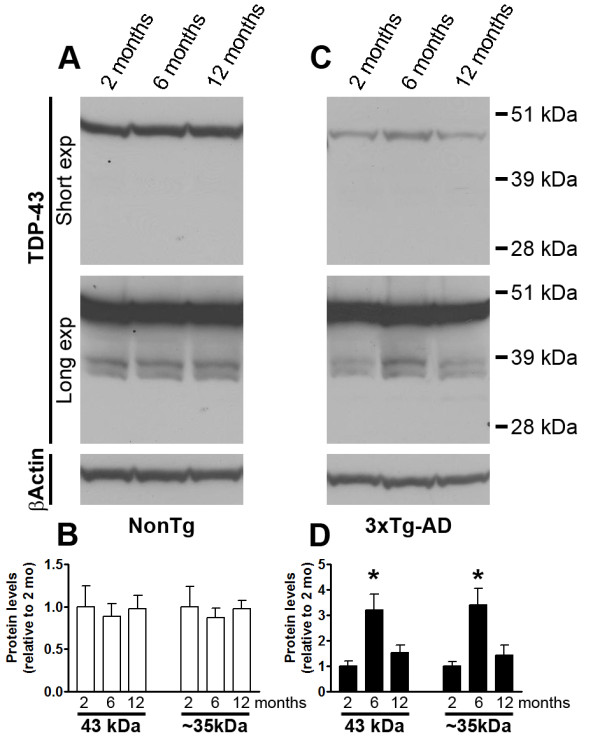
**Age-dependent changes in TDP-43 and TDP-35 levels**. **(A) **Representative Western blots of proteins from the low salt fraction extracted from 2-, 6- and 12-month-old NonTg mice and probed with a polyclonal antibody raised against TDP-43 antibody. A longer exposure time was necessary to see the less abundant low molecular weight fragments. **(B) **Quantitative analysis of the blots shows that the levels of TDP-43 and TDP-35 were similar across the three different ages analyzed (n = 6/time-point). **(C) **Representative Western blots of proteins from the low salt fraction extracted from 2-, 6- and 12-month-old 3×Tg-AD mice and probed with a polyclonal antibody raised against TDP-43 antibody. **(B) **Quantitative analysis of the blots shows that the levels of TDP-43 and TDP-35 were significantly higher at 6 months of age (n = 6/time-point). Abbreviation: exp = exposure. Data are presented as ± SEM and analyzed by one way ANOVA.

The accumulation of TDP-43 has been linked to FTLD-U and ALS [29]. TDP-43 also accumulates in Parkinson's disease, dementia with Lewy bodies, and in 30% of AD cases [34-37,39]. In AD brains, TDP-43 seems to prevalently accumulate in the brain areas more susceptible to Aβ and tau pathology such as hippocampus, amygdala, and in selected cortical regions [40]. However, the relation between TDP-43, Aβ and tau is not known. To investigate the link between Aβ, tau and TDP-43, we first measured the steady-state levels of TDP-43 and its C-terminal fragments in the low salt fraction (see material and methods) of proteins extracted from in 2-, 6-, and 12-month-old 3×Tg-AD and NonTg mice (n = 6/genotype/time-point). We found that in the brains of 2-month-old mice, the steady-state levels of TDP-43 and the ~35 kDa C-terminal fragment (herein referred to as TDP-35), which can be detected by overexposing the blots, were similar between 3×Tg-AD and age- and gender-matched non transgenic (NonTg) mice (Figure 1A-B). In contrast, the brains of 6-month-old 3×Tg-AD mice had significantly higher levels of TDP-43 and TDP-35 than the age- and gender-matched NonTg mice (Figure 1C-D). TDP-43 and TDP-35 levels were again similar between 3×Tg-AD and NonTg mice at 12 months of age (Figure 1E-F). The higher levels of TDP-43 and TDP-35 in 6-month-old 3×Tg-AD mice coincided with the onset of Aβ and tau pathology [38,41]. The difference in TDP-43 levels between 3×Tg-AD and NonTg mice at 6 months of age might be due to a decrease in TDP-43 levels in the brains of 6-month-old 3×Tg-AD mice or to an increase in TDP-43 levels in 6-month-old NonTg mice. To discriminate between these two possibilities, protein extracted from 2-, 6-, and 12-month-old NonTg mice were run on the same blot. We found that in NonTg mice, the levels of TDP-43 and TDP-35 were similar across the three different time points (Figure 2A-B). In contrast, our results indicated that in the 3×Tg-AD mice the levels of TDP-43 and TDP-35 were significantly higher at 6 months of age, compared to 2 and 12 months of age (Figure 2C-D). Taken together, these data indicate that the higher levels of TDP-43 and TDP-35 in the brains of 6-month-old 3×Tg-AD mice compared to NonTg mice are due to an increase of these proteins in the 3×Tg-AD mice as a function of age and not to a decrease of the levels of TDP-43 and TDP-35 in the brains of NonTg mice.

**Figure 3:**
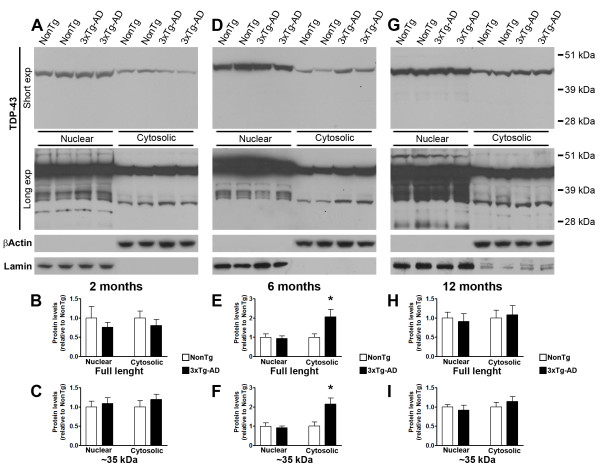
**Age-dependent changes in TDP-43 and TDP-35 cellular localization**. **(A) **Representative Western blots of proteins from the cytosolic and nuclear fractions extracted from 2-month-old NonTg and 3×Tg-AD mice and probed with the a polyclonal anti-TDP antibody. A longer exposure was necessary to see the less abundant low molecular weight fragments. **(B-C) **Quantitative analysis of the blots shows that at 2 months of age the nuclear and cytosolic levels of TDP-43 and TDP-35 were not statistically significant between 3×Tg-AD and NonTg mice (n = 6/genotype). **(D) **Representative Western blots of proteins from the cytosolic and nuclear fractions extracted from 6-month-old NonTg and 3×Tg-AD mice and probed with a polyclonal anti-TDP antibody. **(E-F) **Quantitative analysis of the blots shows that at 6 months of age the nuclear levels of TDP-43 and TDP-35 were not statistically significant between 3×Tg-AD and NonTg mice (n = 6/genotype). In contrast, the cytosolic levels of TDP-43 and TDP-35 were significantly higher in the 3×Tg-AD mice compared to NonTg mice (p = 0.03 and p = 0.01, respectively). **(G) **Representative Western blots of proteins from the cytosolic and nuclear fractions extracted from 12-month-old NonTg and 3×Tg-AD mice and probed with a polyclonal anti-TDP antibody. **(H-I) **Quantitative analysis of the blots shows that at 12 months of age the nuclear and cytosolic levels of TDP-43 and TDP-35 were not statistically significant between 3×Tg-AD and NonTg mice (n = 6/genotype). Abbreviation: exp = exposure. Data are presented as ± SEM and analyzed by t-test analysis.

A neuropathological feature of TDP-43 proteinopathies is the mislocalization of TDP-43, from its predominantly nuclear location into the cytosol, where it accumulates [29] To determine whether there is mislocalization of TDP-43 and TDP-35 in the brains of the 3×Tg-AD mice, we extracted nuclear and cytosolic proteins from the brains of 2-, 6-, 12-month-old 3×Tg-AD and NonTg mice (n = 6/genotype/time-point). Using Western blot analysis, we found that at 2 and 12 months of age, the levels of TDP-43 and TDP-35 were not statistically different between 3×Tg-AD and NonTg mice in the nuclear and cytosolic fractions (Figure 3A-C, G-I). In contrast, at 6 months of age, the levels of cytosolic TDP-43 and TDP-35 were significantly higher in the 3×Tg-AD mice compared to age- and gender-matched NonTg mice (Figure 3D-F). Unexpectedly, we found that the nuclear levels of TDP-43 and TDP-35 were not statistically different between NonTg and 3×Tg-AD mice (Figure 3D-F), suggesting that the increase of the levels in the cytosolic fraction is not simply due to a redistribution of TDP-43 from the nucleus into the cytoplasm. As expected, the levels of TDP-43 and its C-terminal fragments were constantly higher in the nuclear fraction compared to the cytosolic fraction (Figure 3A, D, G). Moreover, in the cytosolic fraction, at all ages and independent of the genotype, the predominant species was TDP-35. In the nuclear fraction there were bands with different molecular weights, indicating more TDP-43 species. Future studies are needed to understand the nature of these fragments.

**Figure 4:**
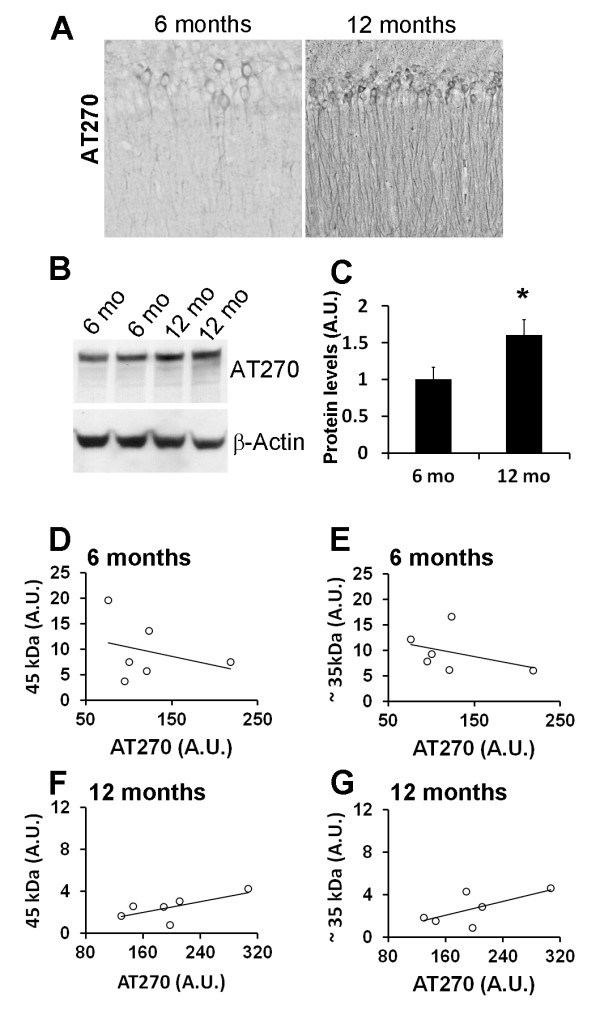
**TDP-43 and TDP-35 levels do not correlate with tau phosphorylated at Thr181**. **(A) **Representative microphotographs of 3×Tg-AD brain sections depicting hippocampal CA1 pyramidal neurons stained with the anti-tau antibody AT270, which recognize tau phosphorylated at Thr181 (n = 6/age group). (B) Representative Western blots of proteins extracted from 6- and 12-month old 3×Tg-AD mice probed with the AT270 antibody. β-actin is used as a loading control. **(C) **Quantitative analysis of the blots shows that the steady-state levels of AT270 were significantly higher in the brains of 12-month-old 3×Tg-AD mice compared to 6-month-old mice (n = 6/age group; p < 0.05). Data are presented as ± SEM and analyzed by t-test analysis. **(D-E) **Linear regression analysis indicated that the levels of AT270 in the brains of 6-month-old 3×Tg-AD mice did not correlate with the levels of TDP-43 (r^2 ^= 0.09148, p = 0.56) and TDP-35 (r^2 ^= 0.1491, p = 0.45). **(F-G) **Similarly, no correlation between AT270 and TDP-43 (r^2 ^= 0.4644, p = 0.14) and TDP-35 (r^2 ^= 0.4469, p = 0.15) levels was observed at 12 months of age.

Tau pathology is another hallmark feature of AD and develops in the 3×Tg-AD mice as a function of age [38,42]. In the 3×Tg-AD mice tau first accumulates in the somatodendritic compartment, and as the mice age, it becomes hyperphosphorylated and eventually aggregates to form NFTs [38,42]. Phosphorylation at Thr181 (recognized by the anti-tau antibody, AT270) is one of the earliest manifestations of tau pathology in the 3×Tg-AD mice and occurs at 6 months of age (Figure 4A). As the mice age, AT270 immunoreactivity increases (Figure 4A), and the age-dependent increase is also detected by Western blot (Figure 4B-C). We next sought to determine whether there is a relation between AT270, TDP-43 and TDP-35. We focused on the AT270 epitope because in the 3×Tg-AD mice tau phosphorylation at Thr181 correlates with cognitive performance (e.g., [43]). Linear regression analysis indicated that the levels of AT270 in the brains of 6-month-old 3×Tg-AD mice do not correlate with the levels of TDP-43 and TDP-35 (Figure 4D-E; r^2 ^= 0.09148, p = 0.56; and r^2 ^= 0.1491, p = 0.45, respectively). Neither was a correlation between AT270 and TDP-43 and TDP-35 levels observed at 12 months of age (Figure 4F-G; r^2 ^= 0.4644, p = 0.14; and r^2 ^= 0.4469, p = 0.15). The lack of correlation between TDP-43 and tau is consistent with data showing that TDP-43 deposits in AD brains do not co-localize with NFTs or dystrophic neuritis [37].

**Figure 5:**
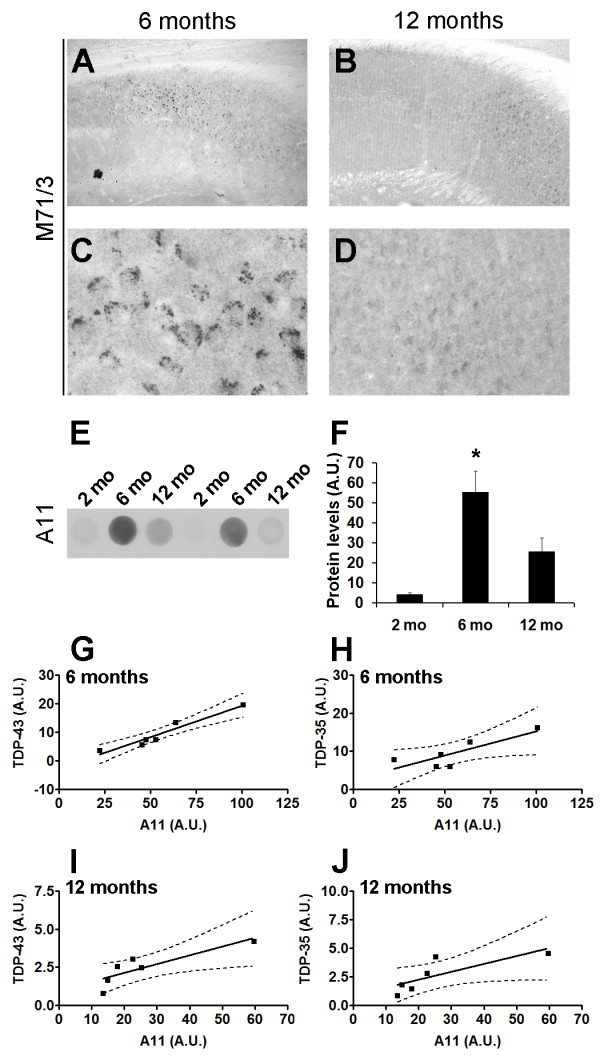
**Aβ oligomers correlate with TDP-43 and TDP-35**. (**A-D) **Representative microphotographs showing 3×Tg-AD hippocampal sections stained with the antibody M71/3, which is specific for Aβ oligomers. Panels C and D show higher magnification views of panels A and B, respectively (n = 6/age group). **(E) **Representative dot blots of protein extracted from 2-, 6-, and 12-month-old 3×Tg-AD mice and probed with the antibody A11, which is specific for Aβ oligomers. **(F) **Quantitative analysis of the dot blots shows that at 6 months of age, A11 levels were significantly higher compared to 2-month-old mice (p < 0.001) and 12-month-old mice (p < 0.05). **(G-H) **Linear regression analysis indicated that the levels of A11 in the brains of 6-month-old 3×Tg-AD mice significantly correlated with the levels of TDP-43 (r^2 ^= 0.9330, p = 0.001) and TDP-35 (r^2 ^= 0.6749, p = 0.045). **(I) **Similarly, the levels of A11 in the brains of 12-month-old 3×Tg-AD mice significantly correlated with TDP-43 (r^2 ^= 0.7259, p = 0.031). **(J) **A strong trend was observed when analyzing A11 and TDP-35 levels in 12-month-old 3×Tg-AD mice (r^2 ^= 0.6277, p = 0.06). The dotted lines in panels G-J represent 95% confidence intervals.

Evidence indicates a primary role for Aβ oligomers in AD pathogenesis [12]. In the 3×Tg-AD mice, Aβ oligomerization starts intraneuronally, and a strong immunoreactivity can be detected with the M71/3 antibody, which is specific for low molecular weight Aβ oligomers (Figure 5A, C; [44,45]). As the mice age, however, the intraneuronal M71/3 immunoreactivity decreases (Figure 5A-D). We have previously shown that this decrease in M17/3 in 12-month-old 3×Tg-AD mice correlates with the appearance of extracellular Aβ plaques [44]. Next, to further analyze the changes in Aβ oligomer levels in the brains of the 3×Tg-AD mice, we performed dot blot experiments using A11, an oligomeric-specific antibody that recognizes high molecular weight Aβ oligomers [46]. Consistent with the M71/3 data, we found that the A11 levels peak at 6 months of age and decrease at 12 months of age (Figure 5E-F). To determine the relation between TDP-43, TDP-35 and Aβ oligomers, we correlated oligomeric Aβ levels obtained by A11 dot blot with TDP-43 and TDP-35 levels in 6- and 12-month-old 3×Tg-AD mice. We found that in 6-month-old 3×Tg-AD mice, A11-positive Aβ oligomers positively correlated with TDP-43 (r^2 ^= 0.9330, p = 0.001; Figure 5G) and TDP-35 (r^2 ^= 0.6749, p = 0.045; Figure 5H). Similarly, we found that A11 levels in 12-month-old 3×Tg-AD mice positively correlate with TDP-43 (r^2 ^= 0.7259, p = 0.031; Figure 5I). A strong trend was also observed when analyzing A11 and TDP-35 levels in 12-month-old 3×Tg-AD mice (r^2 ^= 0.6277, p = 0.06; Figure 5J). Taken together, these data suggest that the increase in TDP-43 in the 3×Tg-AD mice may be due to the increase in soluble Aβ oligomers.

**Figure 6:**
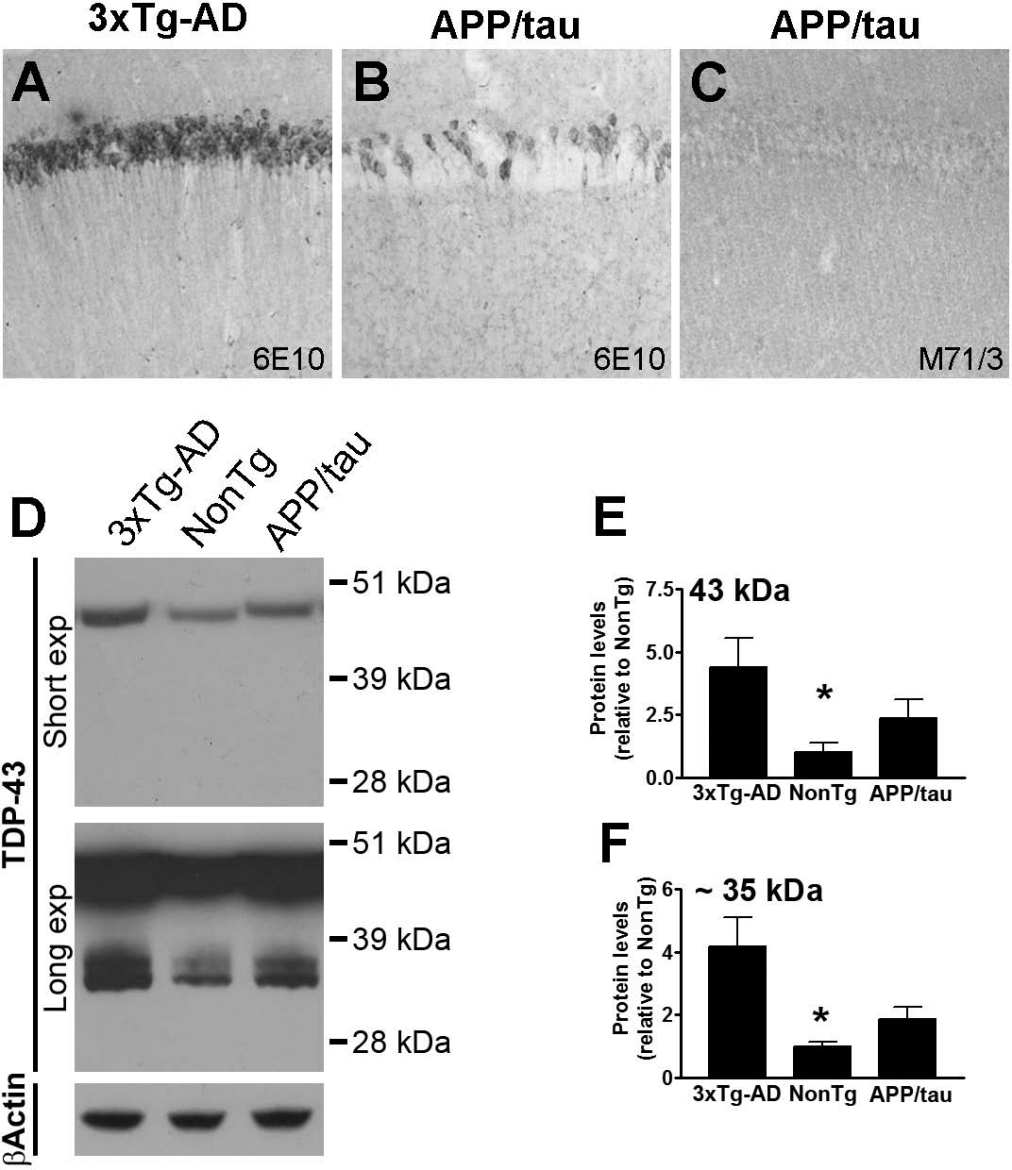
**Reduction of Aβ_42 _levels decreases TDP-43 and TDP-35 levels**. **(A-B) **Representative microphotographs depicting CA1 pyramidal neurons from 6-month-old 3×Tg-AD and APP/tau mice stained with an anti-Aβ antibody (n = 6/group). **(C) **Representative microphotograph depicting CA1 pyramidal neurons from APP/tau mice stained with the anti-oligomeric antibody M17/3. **(D) **Representative Western blots of protein extracted from the brains of 6-month-old NonTg, APP/tau and 3×Tg-AD mice (low salt fraction), probed with a polyclonal anti-TDP-43 antibody. β-Actin is used as a loading control. A longer exposure time was necessary to see the less abundant low molecular weight fragments. (**E-F) **Quantitative analysis of the blots (n = 6/genotype) shows that in APP/tau mice, the steady-state levels of TDP-43 and TDP-35 are significantly lower in APP/tau mice compared to 3×Tg-AD mice (p < 0.05). The levels of TDP-43 and TDP-35 were not statistically significant different between APP/tau and NonTg mice. Data are presented as means ± SEM and were analyzed using one-way ANOVA following by post hoc Bonferroni test to determine individual differences in groups. Abbreviation: exp = exposure.

The strong correlation between Aβ oligomers and TDP-43 levels led us to hypothesize that the buildup of Aβ oligomers maybe the cause underlying the increase in TDP-43 levels in the 3×Tg-AD mice. To test this hypothesis we used a double transgenic mouse model (referred to as APP/tau mice) that we previously generated by replacing the mutant PS1 allele with its wild type counterpart in the 3×Tg-AD mice, thereby obtaining double transgenic mice expressing APP and tau [47]. Because of the M146V mutation in the PS1 gene, the 3×Tg-AD mice accumulate 10 times more Aβ_42 _than Aβ_40 _[38]. Thus, replacing the M146V mutation with its wild type counterpart significantly decreased Aβ_42 _levels [47]. Here we used the APP/tau mice to determine the effects of preventing Aβ oligomers accumulation on TDP-43 levels. At 6 month of age, the APP/tau mice show a significant reduction in intraneuronal Aβ immunoreactivity compared to age- and gender-matched 3×Tg-AD mice (Figure 6A, B). Notably, the APP/tau mice did not show any M71/3 immunoreactivity (Figure 6C). To determine the effect of preventing Aβ accumulation on TDP-43, we measured the steady-state levels of TDP-43 and TDP-35 in the low salt fraction of proteins extracted from the brains of the APP/tau mice by Western blot (Figure 6D). We found that the levels of TDP-43 and TDP-35 were significantly lower in the brains of the APP/tau mice compared to 3×Tg-AD mice (Figure 6E-F). Notably, the levels of TDP-43 and TDP-35 were not significantly different between APP/tau and NonTg mice. Taken together, the results presented here strongly argue of a causal relation between the build-up of Aβ oligomers and the increase in TDP-43 levels.

## Discussion

In addition to representing the major pathological protein that accumulates in CNS inclusions characterizing ALS and FTLD-U, TDP-43 positive inclusions have been found in ~30% of AD cases [34,37,39]. Specifically, the accumulation of low molecular weight C-terminal fragments has been reported in human AD patients [48]. Notably, these fragments may play a primary role in the disease pathogenesis as their expression in vitro is sufficient to cause TDP-43 mislocalization [31-33]. The clinical significance of TDP-43 accumulation in AD and its relation with the two neuropathological hallmarks of AD (Aβ and tau) is not understood. In this study, we elucidate this relation using an animal model of AD. Our results indicate that in the brains of the 3×Tg-AD mice the levels of full length TDP-43 and its ~35 kDa C-terminal fragment change as a function of age and Aβ oligomer levels. Notably, we found that TDP-43 and TDP-35 levels significantly correlated with Aβ oligomers, thereby suggesting a possible relation between Aβ and TDP-43. Toward this end, we found that TDP-43 and TDP-35 levels were higher in 6-month-old 3×Tg-AD mice compared to age-matched NonTg mice, but not at 12 months of age. Previously we showed that Aβ oligomers levels in the 3×Tg-AD mice peak at 6 months of age and are significantly lower at 12 months of age [44], which is consistent with the hypothesis that the increased TDP-43 levels in 6-month-old 3×Tg-AD mice may be due to high levels of Aβ oligomers. Indeed, we show that genetically preventing Aβ_42 _accumulation in the 3×Tg-AD mice is sufficient to decrease TDP-43 levels, further supporting an interaction between Aβ and TDP-43.

It is widely accepted that Aβ oligomers play a central role in AD pathogenesis [17-19]. Toward this end, it has been shown that low concentrations of Aβ oligomers can kill neurons, impair LTP, and lead to cognitive decline [49-55]. Aβ oligomers have been shown to interact with several signaling transduction pathways [56-61]. Although the mechanism underlying the Aβ-mediated accumulation of TDP-43 in the 3×Tg-AD mice remains to be elucidated, it is tempting to speculate that alterations in signaling transduction pathways due to the build-up of Aβ oligomers may be responsible for TDP-43 accumulation and misprocessing. For example, caspase 3 and 7 can cleave TDP-43 and lead to the accumulation of TDP-43 fragments [62]. Notably, elevated caspase 7 mRNA levels have been reported in AD brains [63], and, more specifically, there is evidence that Aβ oligomers can increase caspase activity [64-66]. This is consistent with data showing that TDP-43 can be cleaved, in a caspase dependent manner, to generate TDP-43 C-terminal fragments [62]. Thus, it is tempting to speculate that an Aβ-increase in caspase activity may facilitate the formation of the ~35kDa C-terminal fragment of TDP-43. Additionally, our results show that cytosolic TDP-43 levels are higher in 6-month-old 3×Tg-AD mice compared to age- and gender-matched NonTg mice. Such an increase, however, was not due to a redistribution of TDP-43 from the nucleus into the cytoplasm as we found that nuclear TDP-43 levels were similar between 3×Tg-AD and NonTg mice. It is possible that a reduction in protein turnover may account for the higher levels of TDP-43 and TDP-35 in the 3×Tg-AD mice. Notably, Aβ oligomers have been shown to reduce the activity of the ubiquitin-proteasome-system and autophagy [61,67], two major protein turnover systems that are involved in TDP-43 clearance as independently reported by several laboratories [31,68-70].

Contradicting reports have been published on the relation between TDP-43 and tau pathology. Specifically, in brains from AD patients, more often than not, tau immunoreactivity does not correlate with TDP-43 positive neurons [48], which is consistent with our data showing that TDP-43 and TDP-35 levels did not correlate with phosphorylated tau at Thr181. However, it has been reported that the Braak score for neurofibrillary tau pathology is higher in AD cases with TDP-43 immunoreactivity [48]. To complicate this apparent contradiction are the data showing that in dementia with Lewy bodies, a disease also characterized by tau accumulation, TDP-43 immunoreactivity is not related with Braak neurofibrillary tau pathology [48]. Although we found that in the 3×Tg-AD mice, TDP-43 levels did not correlate with tau phosphorylated at Thr181, further studies are needed to establish whether TDP-43 levels will change in relation to NFT, as suggested by some studies with human brains [34,48], or with total tau levels. Specifically, the latter could not be addressed using the 3×Tg-AD mice, where the tau transgene does not change as a function of age and its steady-state level reflect the promoter activity. Thus any attempt of correlating total tau to TDP-43 would have been artificial. Furthermore, a report by Amador-Ortiz and colleagues shows that in some selective regions of AD brains, TDP-43 deposits correlate with phosphotau, whereas in other regions within the same brain, no correlation was found [34]. This apparent discrepancy could arise from the sample preparation; indeed, the data presented here from the 3×Tg-AD mice where obtained from whole brain homogenize and compare the levels of soluble tau and soluble TDP-43 and TDP-35. Finally, the relationship between other phosphotau epitopes and TDP-43 remains to be determined.

## Conclusions

The data presented here provide compelling evidence that in the brain of the 3×Tg-AD mice, the accumulation of soluble Aβ oligomers may be responsible for the increase in the steady-state levels of TDP-43 and TDP-35. It should be noted, however, that the biochemical profile of TDP-43 detected in the 3×Tg-AD mice was different from that believed pathogenic in FTLD-U [29], suggesting that in these mice changes in TDP-43 levels do not play a role in the AD-like phenotype developed by these mice. Further studies will be needed to elucidate whether TDP-43 plays a clinical role in AD pathogenesis.

## Materials and methods

### Mice

The generation of the 3×Tg-AD and APP/tau mice was previously described [38,47]. Briefly, the 3×Tg-AD mice were generated by co-injecting two different transgenes encoding human APPswe and human tau P301L, both under the control of the Thy1.2 promoter, into single-cell embryos harvested from homozygous mutant PS1M146V knock-in mice (PS1-KI). The APP/tau mice were generated crossing homozygous 3×Tg-AD mice with NonTg mice to replace the mutant PS1 allele with its wild type counterpart.

### Immunohistochemistry

Mice were sacrificed by CO_2 _asphyxiation and their brains rapidly removed and dropped fixed for 48 hours in 4% paraformaldehyde. Free-floating sections (50 μm thick) were obtained using a vibratome slicing system (Leica VT1200S, Germany) and stored in 0.02% sodium azide in PBS. Following two washes with TBS, endogenous peroxidase activity was quenched for 30 minutes in 3% H_2_O_2_. For epitope exposure sections were next incubated in 90% formic acid for 7 minutes, followed by tree additional washes in TBS (100 mM Tris pH 7.5; 150 mM NaCl). The proper primary antibody was applied overnight at 4°C. Sections were washed 3 times in TBS and then incubated with the suitable secondary antibody for 1 hour at room temperature. Sections were then developed with diaminobenzidine (DAB) substrate using the avidin-biotin horseradish peroxidase system (Vector Labs, Burlingame, CA).

### Protein extraction

Following CO_2 _asphyxiation, brains were extracted and frozen in dry ice. To obtain the low and high salt fractions, brains were homogenized with a power homogenizer in 1 ml of low salt buffer (10 mM Tris pH7.5, 5 mM EDTA, 1 mM DTT, 10% Sucrose) in the presence of protease inhibitors. Samples were then centrifuged at 14,400 rpm for 30 minutes at 4°C. The supernatant was stored at -80°C as low salt fraction. To obtain the cytosolic and nuclear fractions, brains were washed in PBS and then homogenized with a dounce homogenizer with 2 ml of solution A (10 mM Hepes pH7.9, 10 mM KCL, 0.1 mM EDTA, 0.1 mM EGTA, 1 mM DTT) in the presence of protease inhibitors. After 5 initial strokes, 0.5% of NP40 was added, and the brain was further homogenized with 5 additional strokes. Subsequently, the solution was kept in ice for 10 minutes and centrifuged 1 minute at 11,000 rpm. The supernatant was removed and stored at -80°C as cytosolic fraction. The pellet was re-suspended in 250 μl of Solution B (20 mM Hepes pH7.9, 400 mM Nacl, 1 mM EDTA, 1 mM EGTA, 1 mM DTT) in the presence of protease inhibitors and placed in ice for 15 minutes. Finally, the tubes were centrifuged 5 minutes at 11,000 rpm and the supernatant was stored at -80°C as nuclear fraction.

### Western blot and dot blot

Proteins were resolved using precast SDS/PAGE gels (Invitrogen, Carlsbad, CA) under reducing conditions and transferred to a nitrocellulose membrane. The membrane was incubated in a 5% solution of non-fat dry milk in T-TBS (0.02% Tween 20, 100 mM Tris pH 7.5; 150 nM NaCl) for 1 hour at 20°C. The membrane was then incubated in the proper primary antibody at 4°C overnight. The blots were washed in T-TBS for 20 minutes and incubated at 20°C with the appropriate secondary antibody for 1 hour. After a final 20-minute wash in T-TBS, blots were developed for 5 minutes with Super Signal (Pierce, Rockford, IL), washed and exposed. For dot-blots, proteins were applied in a nitrocellulose membrane and air dried. Membranes were resolved as described above.

### Antibodies

The following antibodies were used in this study: AT270 (Pierce, Rockford, IL) anti-β-actin (Sigma, St. Louis, MO), rabbit anti human TARDBP polyclonal antibody (ProteinTech Group, Chicago, IL), A11 (a gift from Dr. Charles Glabe, University of California, Irvine), M71/3 (a gift from Dr. William Klein, Northwestern University, Evanston, IL).

### Statistical evaluation

The data were subsequently analyzed by ANOVA or t-test comparison as detailed in the figure legends, using Graphpad Prism software (Graphpad Prism Inc., San Diego, CA).

## Competing interests

The authors declare that they have no competing interests.

## Authors' contributions

AC designed the study, executed most of the experiments and analyzed the data. AM executed some of the Western blots. SO designed the study, analyzed the data and wrote the paper. All authors read and approved the final manuscript.
